# Open-Environment Robotic Acoustic Perception for Object Recognition

**DOI:** 10.3389/fnbot.2019.00096

**Published:** 2019-11-22

**Authors:** Shaowei Jin, Huaping Liu, Bowen Wang, Fuchun Sun

**Affiliations:** ^1^State Key Laboratory of Reliability and Intelligence of Electrical Equipment, Hebei University of Technology, Tianjin, China; ^2^Key Laboratory of Electromagnetic Field and Electrical Apparatus Reliability of Hebei Province, Hebei University of Technology, Tianjin, China; ^3^Department of Computer Science and Technology, Tsinghua University, Beijing, China

**Keywords:** open environment, interactive perception, objects in containers, acoustic features, object recognition, kernel k nearest neighbor

## Abstract

Object recognition in containers is extremely difficult for robots. Dynamic audio signals are more responsive to an object's internal property. Therefore, we adopt the dynamic contact method to collect acoustic signals in the container and recognize objects in containers. Traditional machine learning is to recognize objects in a closed environment, which is not in line with practical applications. In real life, exploring objects is dynamically changing, so it is necessary to develop methods that can recognize all classes of objects in an open environment. A framework for recognizing objects in containers using acoustic signals in an open environment is proposed, and then the kernel k nearest neighbor algorithm in an open environment (OSKKNN) is set. An acoustic dataset is collected, and the feasibility of the method is verified on the dataset, which greatly promotes the recognition of objects in an open environment. And it also proves that the use of acoustic to recognize objects in containers has good value.

## 1. Introduction

With the development of intelligent robots and artificial intelligence, the demand for intelligent service robots in society is increasing. For example, intelligent service robots can take care of the elderly. But intelligent service robots live with older people and must have the same perceptual abilities as people, such as being able to see, touch, or hear what is happening in the world around them. These perceptual abilities will enable robots to perform various tasks, among which object recognition is one of the most common and important tasks. To accomplish this task, intelligent service robots can be equipped with multiple types of sensors, each of which can reflect the properties of an object from different aspects. Currently, the most widely used sensor is the camera, because a large amount of information about an object can be obtained from a single image. Therefore, the study of vision in object recognition has attracted great attention. For example, color (Forero et al., 2018), texture (Kaljahi et al., 2019), and appearance (Liu et al., 2010) can be classified by visual images. However, the vision is sometimes affected by factors such as illumination, object color, occlusion, and posture of objects. It is difficult to find some intrinsic properties of objects, such as softness, stiffness, and material properties.

In addition, the force sensor responds to some object properties according to the contact force when contacting the object, for example, by directly contacting the object for shape recognition (Luo et al., 2016), category recognition (Gandarias et al., 2018), material retrieval (Strese et al., 2017), and surface roughness recognition (Yi et al., 2017). By simply touching an object that only recognizes the object being touched, it is impossible to perceive objects in the container, such as food in a kitchen container or medication in a bottle. Interactive perception is a common human exploration strategy. If humans cannot recognize objects by vision and touch, they will take different interactions to obtain information about other sensory channels. For example, shaking a hollow object produces auditory information that can determine whether the object is empty or full, what material is contained inside, and how much material is inside.

Sound-based object recognition is less studied than object recognition based on visual and tactile information. Despite this, hearing is as important as touch and vision, especially in the dark or dangerous environments. Hearing has a unique advantage. Sound and structural vibration signals provide a rich source of information for manipulating objects. Humans use this feedback to detect mechanical events and estimate the state of the manipulated object. Hearing allows us to infer events in the world that often go beyond the scope of other sensory modes. Studies have shown that humans are able to extract the physical properties of objects and distinguish between different types of events from the sound produced (Grassi, 2005; Beran, 2012). There has been some research that uses sound for positioning (Brichetto et al., 2018), cup material recognition (Griffith et al., 2012), pouring height (Liang et al., 2019), family event recognition (Chang et al., 2016; Do et al., 2016), object material recognition (Neumann et al., 2018), contact position recognition and qualitative size of contact force (Zöller et al., 2018).

The ability to sense and process vibrations during interactive contact with an object will allow the robot to detect anomalies in the interaction process and perform object recognition based on the vibration signals. The interaction between objects produces a vibration signal that propagates through the air and can be perceived by the acoustic sensor. The cost of collecting and processing vibration feedback is relatively low relative to other sensory modes (such as vision). Object recognition is a fundamental skill that occurs during the early development of human beings. When interacting with objects, it will try a variety of interactive ways to complete the task of recognizing objects. At the same time, when contacting an object, it is necessary to detect whether the object in contact has been encountered before, that is, the object being explored is an object of unknown classes. Making a robot separate objects of unknown classes from objects of known classes like a human, then relearn the information of the unknown classes. Therefore, it is very important for robots to develop a system that can recognize objects of unknown classes and reach the recognition of all objects by continuously learning the properties of unknown classes. The main contributions of this paper are as follows:

1. A framework for recognizing objects in containers using acoustic signals in an open environment is proposed.
2. The kernel k nearest neighbor algorithm (OSKKNN) in an open environment is proposed to solve the problem of recognizing all class objects in an open environment.
3. The acoustic dataset was collected using the UR5 arm with the microphone and verified the effectiveness of our method.

The remainder of the paper is organized as follows. In section 2, the related work of collecting sound to recognize objects is reviewed. In section 3, a framework for recognizing objects using sound is introduced, and an OSKKNN algorithm is proposed. Acoustic dataset collection and data analysis and processing are in section 4. Section 5 conducts experiments and experimental analysis. Finally, the paper is concluded in the last section.

## 2. Related Work

It is very challenging to recognize objects contained in containers and objects of different weight in containers. These studies are few. When the visual and tactile constraints are limited, the perceptual information generated by the simple static contact is also difficult to recognize objects in containers. We naturally use dynamic contact methods to obtain information about objects. Berthouze et al. (2007) and Takamuku et al. (2008) pointed out that dynamic contact (shaking) is more conducive to recognizing objects than static contact (grasping), which is not easily affected by the shape, size and color of objects. When shaking the object, it will produce the sound signal of vibration, which can be collected by a microphone. There is related research on the use of shaking to collect sound signals (Nakamura et al., 2009, 2013; Araki et al., 2011; Taniguchi et al., 2018).

Interactive contacts with objects in different ways and acquisition of sound information to recognize objects are studied as follows: Clarke et al. (2018) used the actions of shaking and pouring to obtain the sound signal of the granular object and combined this with deep learning to recognize five different types of granular objects. Luo et al. (2017) used a pen to hit objects to collect sound information, and used the Mel-Frequency Cepstral Coefficients (MFCCs) and its first and second differential as features; stacked denoising autoencoders are applied to train a deep learning model for object recognition. Sinapov et al. (2009) and Sinapov and Stoytchev (2009) used humanoid robots to perform five different interactive behaviors (grasp, shake, put, push, knock) on 36 common household objects (such as cups, balls, boxes, cans, etc.) and used the k nearest neighbor algorithm (KNN), support vector machine algorithm (SVM) and unsupervised hierarchical clustering to recognize objects. Sinapov et al. (2011) collected the joint torque of the robot and sound signals, and combined with the k nearest neighbor algorithm (KNN) to recognize 50 common household objects.

Sinapov et al. (2014) and Schenck et al. (2014) used ten kinds of interactions (such as grasp, shake, push, lift, etc.) to detect four classes of large-particle objects of three colors and three weights. They not only learn categories describing individual objects, but also learn categories describing pairs and groups of objects, and the C4.5 decision tree algorithm is used to classify and the robot learns new classes based on the similarity measurement method. Chen et al. (2016) tested four kinds of containers (glass, plastic, cardboard and soft paper) with 12 kinds of objects and collected sound signals through shaking using Gaussian naive Bayes algorithm (GNB), support vector machine algorithm (SVM) and K-means clustering algorithm (K-Means) to classify and recognize objects. And it is proved that the sound of shaking can be used for object recognition in many places such as shopping malls, workshops and home. Eppe et al. (2018) used a humanoid robot to perform auditory exploration of a group of visually indistinguishable plastic containers filled with different amounts of different materials, proving that deep recursive neural structures can learn to distinguish individual materials and estimate their weight.

The above research focuses on object recognition in multiple occasions and closed environments and does not pay attention to recognizing objects in specific applications and open environments. There are some studies on different recognition in the open environment, such as the following literature (Bendale and Boult, 2016; Bapst et al., 2017; Gunther et al., 2017; Moeini et al., 2017; Bao et al., 2018), these studies recognize known classes and detect unknown classes in an open environment, but do not recognize all classes. In the real world, objects touched by robots are constantly changing. How can the robot system be made to be like human beings? When encountering unknown objects, it can be well separated from known objects and relearn relevant knowledge of unknown objects. Therefore, it is very important for robots to develop a systematic framework that can detect objects of unknown classes and recognize all objects through continuous learning of the properties of unknown classes. This paper mainly studies the use of sound to recognize household food objects in containers and focuses on the recognition of all class objects in containers using sound in an open environment.

## 3. Kernel k Nearest Neighbor Method for Acoustic Recognition in Open Environment

The open environment no longer assumes that the test set classes and the training set classes are identical, and the open environment is more in line with the actual process of exploring objects. The open environment points out that the classes of the test set will present classes never seen on the training set. Traditional machine learning is carried out in a closed environment and cannot solve the object recognition problem in the actual open environment. If there are unknown classes in the test set, the objects of these unknown classes will be marked as some classes in the training set by using traditional machine learning, which is not in line with the actual classification recognition process and human learning process. This paper develops a systematic framework for recognizing objects using audio in an open environment.

### 3.1. Open Environment Acoustic Recognition Framework

**Figure 1:**
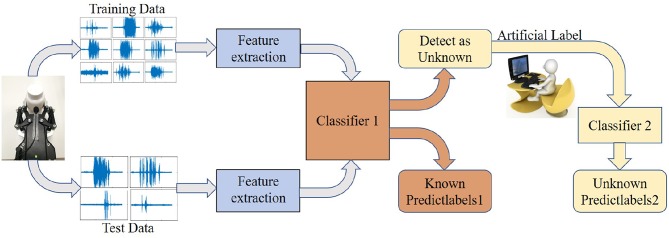
Acoustic recognition framework in open environment.

In the actual learning process, people can recognize the object well when they encounter an object that needs to be recognized. On the other hand, even if the people do not know it they will say that this is an unknown class that they had not seen. If the people want to recognize what this object is, they must recognize it by looking up a book or asking someone who recognizes it to relearn. The system framework is proposed in this paper, which uses audio to recognize objects in an open environment. It is similar to the human learning process. The system framework is shown in Figure 1.

The acoustic recognition process in an open environment is shown in Figure 1. Under this framework, the acoustic data are collected by the robot platform, and the data set is divided into a test set and a training set, but the test set contains classes not seen in the training set. First, acoustic feature extraction is performed on the data, and then the classifier 1 recognizes known object classes of a test set through training a training set, and the classifier 1 detects unknown object classes of a test set. These data of unknown classes are collected and manually labeled. Finally, these data of unknown classes are relearned and trained by the classifier 2. The key part in the acoustic recognition framework is the role of classifier 1. It not only needs to recognize accurately objects of known classes but also needs to detect objects of unknown classes. Only objects with unknown classes can be detected for better relearning and recognizing.

### 3.2. Kernel k Nearest Neighbor in an Open Environment

The k nearest neighbor algorithm is the most mature and simplest classification algorithm, with low training time complexity. It is widely used in various fields, such as text classification (Yong et al., 2009), face recognition (Weinberger et al., 2006), image classification (Zhang et al., 2006; Boiman et al., 2008; Guru et al., 2010), object recognition (Sinapov et al., 2009, 2011), etc. In order to solve different problems, relevant research has been done to improve and optimize the k nearest neighbor algorithm, as in the literature (Weinberger et al., 2006; Zhang et al., 2006; Boiman et al., 2008; Yong et al., 2009; Guru et al., 2010; Kibanov et al., 2018; Liao and Kuo, 2018). This paper uses acoustic properties to recognize objects. The properties of these objects are very close, such as particle size, density, so collected acoustic data are linearly inseparable. If the training test data set has the problem of linear inseparability, then the k nearest neighbor algorithm's similarity measurement effect dependent on distance will become worse and the recognition accuracy will be reduced. To solve this problem, kernel function can be introduced into the k nearest neighbor algorithm to improve the recognition effect of the nearest neighbor algorithm (Yu et al., 2002).

Traditional machine learning is classified and recognized in a closed environment. The open environment recognition problem is a class that does not exist in the training sample in the test sample. It is very difficult to use the kernel k nearest neighbor algorithm in an open environment for this problem. Therefore, based on the kernel k nearest neighbor algorithm, this paper proposes OSKKNN algorithm to recognize objects of all classes in an open environment.

Kernel function includes linear kernel function, polynomial kernel function and radial basis kernel function. Among them, the radial basis kernel function is the most commonly used kernel function, which can map data to infinite dimensions and is a scalar function with radial symmetry. In this paper, the radial basis kernel function is introduced into the k nearest neighbor algorithm.

Definition of radial basis function: *X*_1_ and *X*_2_ represent the eigenvectors of input space, the radial basis kernel function is as follows:

(1) $$\begin{array}{r} K\left(X_{1},X_{2}\right)=\text{exp}\left(-\frac{{\left(\left\|X_{1}-X_{2}\right\|\right)}^{2}}{2\sigma^{2}}\right)=\text{exp}\left(-\gamma {\left(\left\|X_{1}-X_{2}\right\|\right)}^{2}\right) \end{array}$$

Where σ is the hyperparameter of RBF kernel, and the characteristic length-scale of learning samples' similarity is defined, that is, the proportion of the distance between samples before and after feature space mapping in the perspective of weight space (Chang and Lin, 2011; Liao and Kuo, 2018), which can be simplified into a more general form when $\gamma =\frac{1}{2\sigma^{2}}$.

The OSKKNN algorithm is described as follows:

1. Convert training samples and test samples into kernel matrix representations by kernel function;
2. Calculate the distance from the test set sample to each training set sample by the kernel matrix representation;
3. Sort by distance from near to far;
4. Selecting a training set sample of k closest to the current test set sample as a neighbor of the test sample;
5. Count the class frequencies of the k neighbors;
6. Calculate the average value of the nearest k neighbor distances, compare the size of the average value and the threshold value *T*;
7. If it is less than this threshold value *T*, the class with the highest frequency among the k neighbors is the class of the test sample;
8. If it is greater than this threshold value *T*, the test sample is of unknown classes;
9. Collect these unknown class samples and divide them into training set and test set;
10. Use the kernel k nearest neighbor algorithm to train and recognize these unknown objects.

## 4. Acoustic Dataset Collection

**Figure 2:**
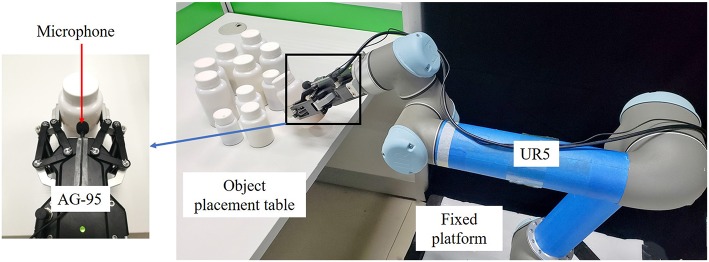
Robot experiment platform.

Our dataset is obtained through the robot experiment platform shown in Figure 2. The robot experiment platform is mainly composed of five parts: Fixed platform, UR5 robot arm (Universal Robots), AG-95 manipulator (DH Robotics), microphone (acquisition frequency 44. 1 kHz, the sound is collected through a standard 35 mm plug into the computer interface, and the sound data are read and saved using the Matlab program) and object placement table. The AG-95 manipulator and UR5 robot arm are connected structurally through a flange and communicate with UR5 through a network wire. Moreover, the grabbing experiment of objects can be easily realized through programming tools equipped with UR5. In order to reduce the impact of environmental noise, we fixed the microphone to the palm of the AG-95 manipulator. During the experiment, the UR5 robot arm drives the AG-95 manipulator to grab the container with different objects on the object placement table according to the planned path, completes the specified shaking action in the air, collects the acoustic signal during the shaking process, and then puts the container back to the original position and returns the UR5 robot arm and AG-95 manipulator to the original position.

### 4.1. Interaction Actions

When the robot interacts with the object, the container is shaken and the object in the container collides with the wall of the container to generate a sound waveform. The time for collecting the contact object is 6 s, and the sampling frequency is 44.1 kHz. In order to test which acquisition method is more suitable for robots and recognition, we use the following four actions, which can be implemented by the programming tool of UR5 robot arm configuration.

**Figure 3:**
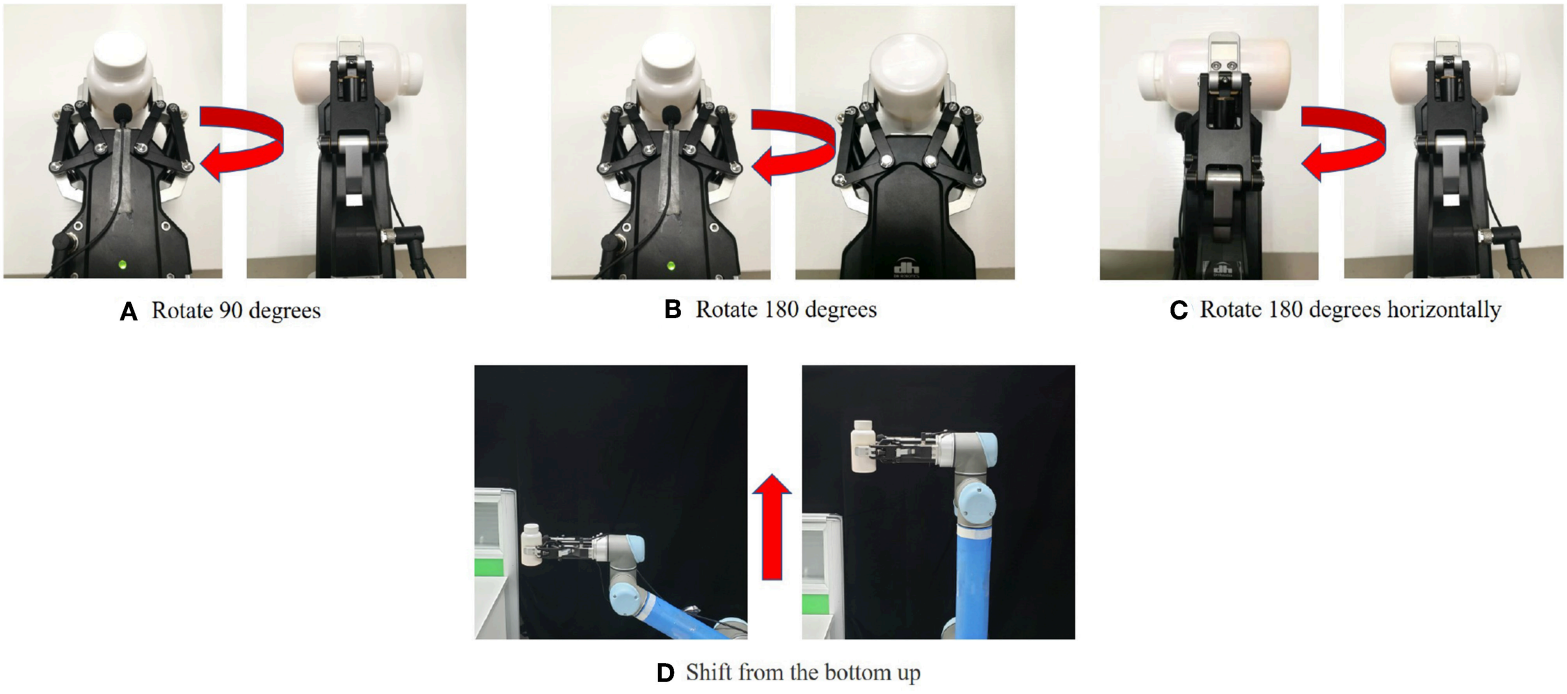
Interaction actions.

1. Rotate 90°: As shown in Figure 3A. Turn clockwise 90° from the direction of the AG-95 manipulator's finger. At the beginning, the container mouth is up before rotating AG-95 manipulator, as shown in the position on the left side of Figure 3A. The container mouth is to the right after rotating AG-95 manipulator, as shown in the position on the right side of Figure 3A. Turn on the sound collection device before rotating, turn off the sound collection device after rotating, and then save the data.
2. Rotate 180°: Same as rotate 90°, except that the angle of rotation is different. Rotate clockwise 180° from the direction of the AG-95 manipulator's finger, as shown in Figure 3B.
3. Rotate 180° horizontally: As shown in Figure 3C. Turn 180° clockwise from the direction of the finger of the AG-95 manipulator. At the beginning, the mouth of the container is on the left before rotating the AG-95 manipulator, as shown in the position on the left side of Figure 3C. The mouth of the container is to the right after rotating the AG-95 manipulator, as shown in the position on the right side of Figure 3C. Turn on the sound collection device before rotating, turn off the sound collection device after rotating, and then save the data.
4. Shift from the bottom up: As shown in Figure 3D. Move parallel from bottom to top in the air. Before moving, the AG-95 manipulator is in the lower position on the left side of Figure 3D, wait to open the sound collection device. After the end of the movement, the position on the right side of Figure 3D is at a higher position. Then turn off the sound collection device and save the data.

### 4.2. Objects Selections

**Figure 4:**
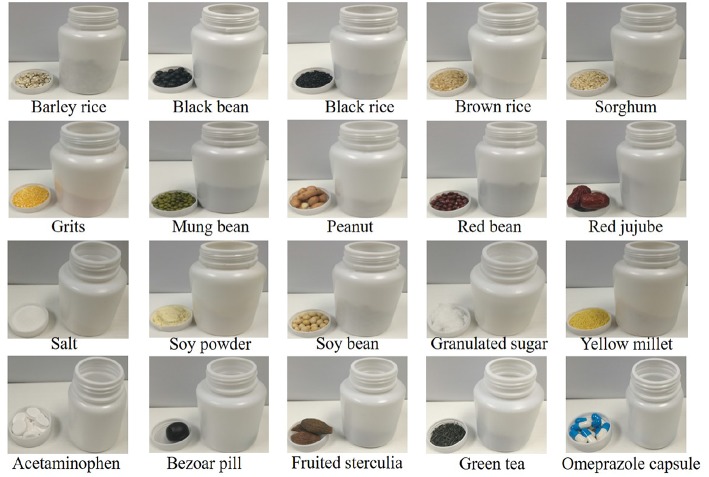
Objects and bottles used.

In order to be applied to household food recognition, 20 kinds of food materials and medicines are selected as shown in Figure 4, and the selected objects are difficult to distinguish. Data acquisition is performed using the interactive methods of section 4.1, and each object is subjected to sound collection 30 times. The sound data are collected on a 16-bit mono at a sampling frequency of 44.1 kHz and saved as a waveform file. The sample data collected by each method are 20 × 30 = 600, and the sample data are collected by the four interaction methods. So, an acoustic dataset of 2,400 samples was established.

Figure 4 shows different objects and corresponding plastic bottles. The plastic bottles used are made of polyethylene. The volume of the plastic bottle is as follows: 200 ml (bottom diameter: 6.1 cm, bottle height: 8.9 cm), 100 ml (bottom diameter: 5.1 cm, bottle height: 5.6 cm), 50ml (bottom diameter: 4.1 cm, bottle height: 4.4 cm). Plastic bottles of different volumes are chosen because the volume of containers required to hold different objects in real life is different.

Figure 4 shows order of the items (weight, volume of the container): barley rice (50 g, 200 ml), black bean (50 g, 200 ml), black rice (50 g, 200 ml), brown rice (50 g, 200 ml), sorghum (50 g, 200 ml), grits (50 g, 200 ml), mung bean (50 g, 200 ml), peanut (50 g, 200 ml), red bean (50 g, 200 ml), red jujube (50 g, 200 ml), salt (50 g, 100 ml), soy powder (50 g, 200 ml), soybean (50 g, 200 ml), white granulated sugar (50 g, 100 ml), yellow millet (50 g, 200 ml), acetaminophen tablet (10 g, 50 ml), bazaar supernatant pill (18 g, 100 ml), fruited sterculia (20 g,100 ml), green tea (12 g, 100 ml) and omeprazole capsule (8 g, 50 ml).

### 4.3. Data Analysis and Processing

**Figure 5:**
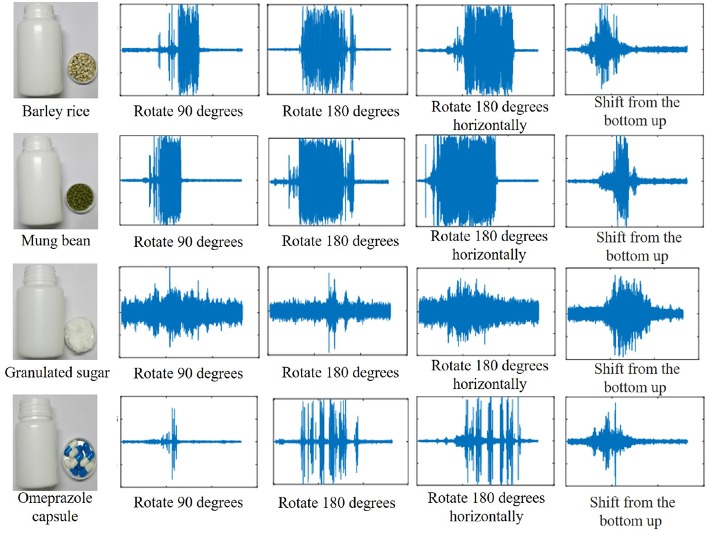
Acoustic waveforms of four kinds of objects (Barley rice, Mung bean, Granulated sugar, Omeprazole capsules) were collected by interactive methods.

The sound signals are collected by objects in the container in different interactive methods. Figure 5 shows four representative objects and collected acoustic signals. By longitudinal comparison, it can be concluded that a sound signal can be used for recognition research. By horizontal comparison, it can be concluded that the sound signals collected by different interactive methods are different, and the recognition effects will be different. In the process of sound collection, a wiener filter needs to be firstly adopted to reduce noise due to the large noise of the environment and UR5 robot arm.

Wiener filtering is a wiener filtering algorithm based on a priori SNR proposed by Scalart and Filho (1996). It is an optimal estimator for stationary processes based on the minimum mean square error criterion. The mean square error between the output of this filter and the desired output is minimal, so it is an optimal filtering system. It can be used to extract signals that are contaminated by stationary noise (Le Roux and Vincent, 2012).

In order to reflect better the properties of the object, it is necessary to propose better features from the noise signal after noise reduction. There are other methods of dimensionality reduction (Li et al., 2017), but the Mel-Frequency Cepstral Coefficients(MFCCs) can not only reduce the data dimension but also the dynamic properties of the sound. The MFCCs are one of the most commonly used features in speech processing. The feature extraction method can also effectively reduce the dimension, thus reducing the computational cost. Related studies have successfully applied the Mel-Frequency Cepstral Coefficients (MFCCs) to speech feature extraction and object recognition, as in the literature (Nakamura et al., 2013; Luo et al., 2017; Eppe et al., 2018). The standard MFCC feature can only propose the static characteristics of the sound (Cao et al., 2017). In order to better reflect the dynamic characteristics of the sound, this paper uses the first-order and second-order different features of the static 12-order MFCCs to obtain the dynamic features of 36-dimensional MFCCs.

## 5. Experiment

### 5.1. Object Recognition in Closed Environment

#### 5.1.1. Comparison of Learning Algorithms and Comparison of Interaction Methods

The classification problem in a closed environment assumes that the training set and the test set have the same classes of objects. In this section, we selected four supervised learning algorithms for comparison, namely the traditional k nearest neighbor algorithm (KNN), the support vector machine algorithm (SVM) of radial basis kernel function (Chang and Lin, 2011), the sparse representation classification algorithm (SRC) (Patel et al., 2011; Pillai et al., 2011), and the kernel k nearest neighbor algorithm (Kernel-KNN) (Yu et al., 2002). We first determined the *K*-values of KNN and Kernel-KNN through experiments. The experimental results were better in the K range of 8–16. We took *K* = 14 in OSKKNN. We used four different interactive methods when collecting data, so we conducted recognition experiments on four data sets. The proportion of the data in the training set and test set is 2:1, and the experimental results are shown in Table 1.

**Table 1:** The recognition accuracy of different interactions and supervised learning methods.

**Acquisition methods**	**KNN%**	**SVM%**	**SRC%**	**Kernel-KNN%**
Rotate 90°	56	62.5	48.5	65.5
Rotate 180°	75	78.5	60.5	82.5
Rotate 180° horizontally	74.5	79.5	75	85.5
Shift from the bottom up	33	34.5	32.5	33.5

Firstly, the influence of the data interaction method on object recognition accuracy is compared with the experimental results in Table 1. The experimental results in Table 1 show that the interactive method of rotating 180° horizontally has a good recognition effect on objects, with the recognition accuracy reaching 85.5%. The recognition effect of rotating 180° is close to that of rotating 180° horizontally, with a difference of 0–5% in recognition accuracy. Therefore, it is appropriate to use the wrist rotation of 180° when the robot collects sound data. By comparing rotate 90° and rotate 180°, it can be inferred that the larger the rotation angle is in the same direction, the more information about object properties is reflected in the collected data, and the higher the recognition accuracy is. Rotate 180° and rotate 180° horizontally, and rotate 90° are rotated around the wrist of the UR5 robot arm. The noise of the UR5 robot arm itself is small, so the data collection can better reflect the attributes of the object. The single joint rotation method can be used to explore the attributes of the object interactively and achieve better recognition effect. The way of shifting from the bottom up, the multi-joint movement of the UR5 robot arm and the loud noise of the UR5 robot arm itself completely cover the sound of the interaction between the AG-95 manipulator and object, and the sound reflecting the object properties cannot be collected. The data set is full of noise, so the object recognition accuracy is very low.

By comparing the four supervised learning algorithms, the experimental results in Table 1 are as follows: the kernel k nearest neighbor algorithm has the best recognition effect and is more suitable for multi-classing. Therefore, the recognition effect after combining the kernel function exceeds the support vector machine algorithm. The k nearest neighbor algorithm combined with the kernel function improves the recognition performance compared with the traditional k nearest neighbor algorithm, and better solves the problem of linear indivisible object recognition. Sparse representation classification was first proposed for image recognition, and the recognition of sound information and small data sets is not good. Therefore, this paper extends the Kernel-KNN algorithm to solve the problem of recognizing unknown class objects in an open environment.

We consider the influence of the kernel function on the KNN algorithm. We compare the linear kernel, the polynomial kernel, the Sigmoid kernel, the rational quadratic kernel, and the radial basis kernel on a horizontally rotated 180° data set. The experimental results are shown in Table 2. Experiments show that the KNN algorithm with radial basis kernel is better.

**Table 2:** Recognition accuracy of different kernel functions.

**Dataset**	**Linear%**	**Polynomial%**	**Sigmoid%**	**Rational quadratic%**	**Radial basis%**
Rotate 180° horizontally	72.5	75	71	85	85.5

#### 5.1.2. Comparison of Weight

When the object is in constant use, the weight is decreasing. For the same object, regardless of the weight, it must recognize the same object. Therefore, we conducted the following experiments, the first interactive method (rotate 180°) is used to collect sound data of 10 objects for verification. The 10 kinds of objects are barley rice, black bean, black rice, brown rice, sorghum, grits, mung bean, red bean, soybean, and yellow millet; the plastic bottles used in these 10 objects are the same plastic bottles as the corresponding ones in section 4.2, but weight 100 g.

**Figure 6:**
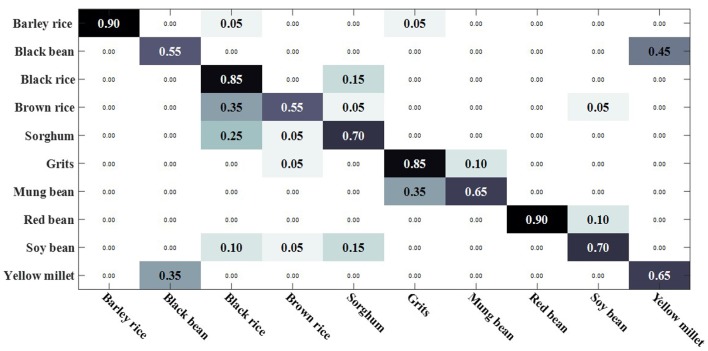
Kernel-KNN recognition confusion matrix of mixed 50 and 100g.

The experimental results are shown in Table 3. It can be concluded from Table 3 that the Kernel-KNN algorithm has a better recognition effect when the object in the plastic bottle is 50 g. This is because the generated sound is larger when the weight of the object in the bottle is 50 g, and it can be inferred that the recognition accuracy of the internal object recognition rate decreases with the increase of weight when the bottle volume is the same. For mixed recognition of 50 and 100 g, the confusion matrix of recognition is shown in Figure 6. Although the recognition effect decreases by 0.5 to 5%, the object in a container can still be well recognized. It proves that no matter what the weight of the object in a container is, the object in the container can also be well recognized by using sound. At the same time, it shows that the sound has a valuable application value for a home intelligent service robot to recognize objects in containers.

**Table 3:** The recognition accuracy of different weights and different supervised learning methods.

**Weight (*g*)**	**KNN%**	**SVM%**	**SRC%**	**Kernel-KNN%**
50	70	72	68	78
100	66	73	50	74
Mixture of 50 and 100	64.5	68	50	73

### 5.2. Object Recognition in Open Environment

Object recognition in an open environment is that the classes of test set and training set are not the same, and the number of classes of the test set is greater than that of the training set. Object recognition in an open environment is more suitable for practical application and the human learning process. Object recognition in an open environment requires two steps. Firstly, samples of known classes are recognized in the open environment, samples of unknown classes are detected and collected in an open environment. Then, these unknown classes are re-learned, which is in line with the continuous learning process of human beings.

#### 5.2.1. Detection of Unknown Class Objects in Open Environment

In the open environment, the known classes are recognized and the unknown classes are detected, and then the objects of the unknown classes are collected. In other words, no matter how many classes the unknown classes are, it is not recognized as long as the unknown classes are detected.

We compared the open set sparse representation classification method for recognizing faces in an open environment (Moeini et al., 2017). This method only recognizes known classes in an open environment, detects unknown classes, and does not recognize unknown classes, so the OSKKNN algorithm is set up in the same way. The experiment was compared on a data set of rotating 180° and rotating 180° horizontally. We set up a training set of 10 classes and test sets of 20 classes (including 10 of the training set).

**Figure 7:**
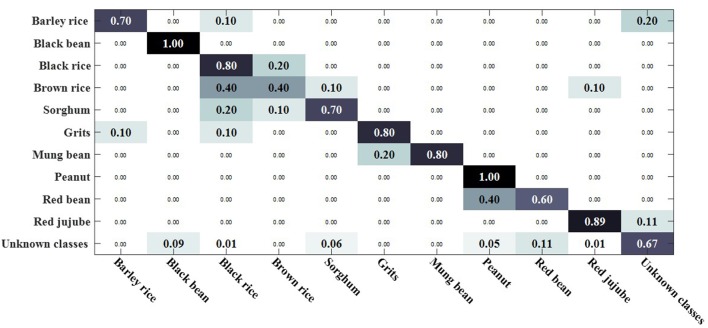
OSKKNN recognition confusion matrix on the data of rotate 180°.

**Figure 8:**
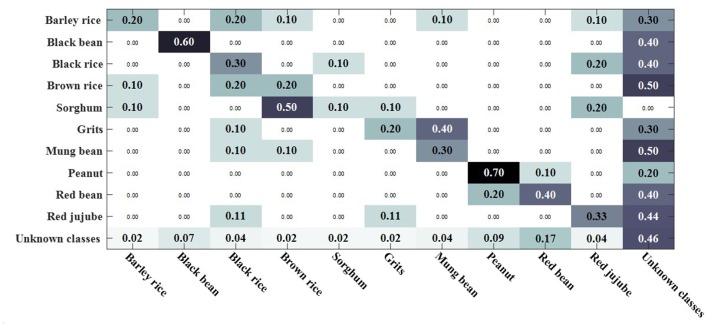
OSSRC recognition confusion matrix on the data of rotate 180°.

**Figure 9:**
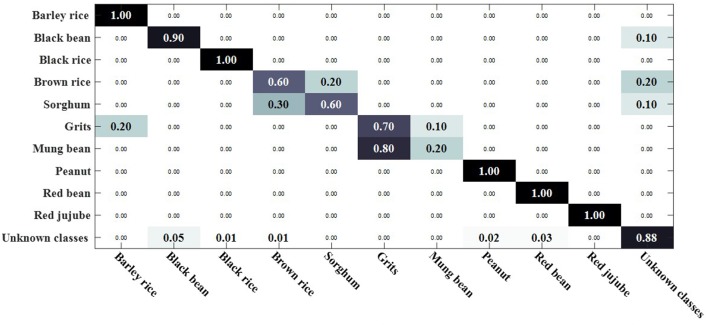
OSKKNN recognition confusion matrix on the data of rotate 180° horizontally.

**Figure 10:**
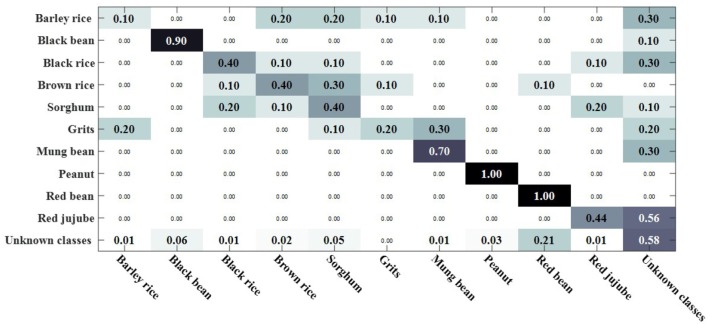
OSSRC recognition confusion matrix on the data of rotate 180° horizontally.

Figures 7, 8 are, respectively, the confusion matrix of OSKKNN and OSSRC on the dataset of rotating 180°. Figures 9, 10 are, respectively, the confusion matrix of OSKKNN and OSSRC on the dataset of rotating 180° horizontally. By comparing Figures 7–10, it can be concluded that OSKKNN has a better effect than OSSRC in separating known classes and unknown classes, and has a better effect in recognizing known classes. Only by separating sample data from unknown classes can you better collect this data and prepare to continue learning these unknown classes. As far as we know, there is no research on the object recognition of unknown classes in an open environment at present.There are only similar studies (Moeini et al., 2017) that recognize objects of known classes in the open environment and detect unknown classes without recognizing objects of unknown classes. OSKKNN can recognize objects of unknown classes detected, and finally recognize objects of all classes.

#### 5.2.2. Recognition of All Class Objects in Open Environment

The threshold value *T* is an important factor affecting the recognition of all class objects in an open environment. A reasonable threshold value *T* setting can successfully separate objects of known classes from objects of unknown classes. The influence of unknown classes on the recognition accuracy of known classes can be reduced and the overall recognition accuracy can be increased only by separating known classes from unknown classes in the test set.

The threshold value *T* selection is different for different dataset thresholds and needs to be determined experimentally. We experimented on rotate 180° data and rotate 180° horizontally data. The number of classes in the experimental training set was randomly selected from 10 classes. The test classes included all classes.

**Figure 11:**
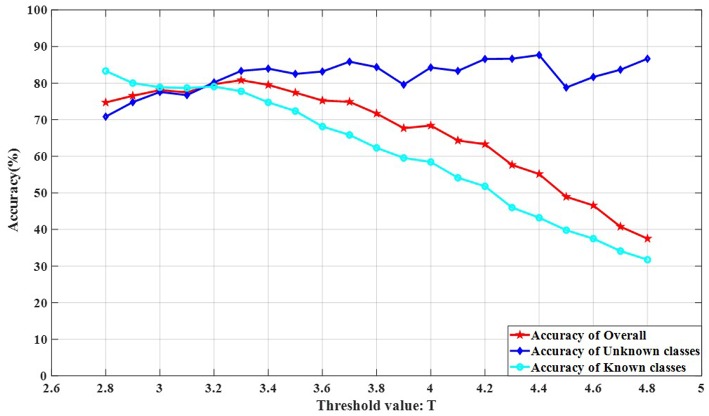
The influence of OSKKNN threshold value *T* on the recognition accuracy of rotate 180° data.

**Figure 12:**
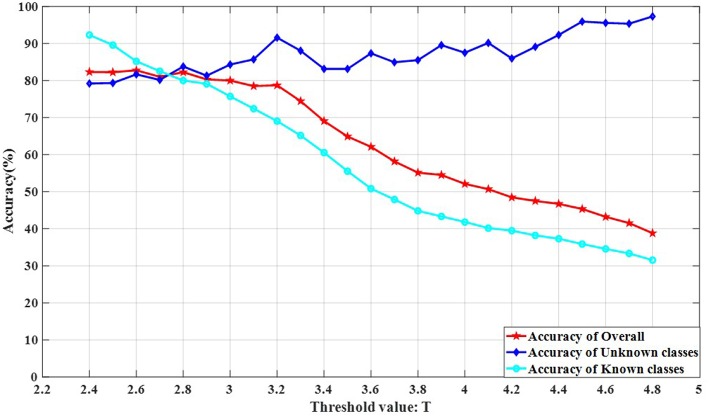
The influence of OSKKNN threshold value *T* on the recognition accuracy of rotate 180° horizontally data.

The experimental results are shown in Figures 11, 12. It can be seen from Figures 11, 12 that the recognition accuracy of the known classes is decreasing as the threshold is increased. This is because the larger the threshold value *T* is, the more samples of unknown classes appear in the known classes test data, the more difficult it is to recognize the samples of known classes. As the threshold value *T* increases, the recognition effect of the unknown classes is better. This is because the larger the threshold value *T* is, the fewer the samples of known classes are in the unknown classes. The recognition accuracy is no longer affected by the sample classes, but only depends on the performance of the classifier 2 (Kernel-KNN). The overall recognition accuracy rate has an inconspicuous upward trend, followed by a significant downward trend. This is because the rising stage, the recognition accuracy rate of known classes decreases slightly, while the recognition accuracy rate of unknown classes has an insignificant rising trend, and the rising range is larger than the falling range of the known classes. The obvious downward trend is because the decline rate of the recognition accuracy rate of known classes is greater than the increase of the unknown classes, and the decline rate of known classes has a greater impact on the overall recognition accuracy rate.

It can be concluded from the experiment that the threshold value *T* of the data set on rotate 180° is in the range of 3.2–3.6, and the overall recognition effect of OSKKNN is better. For the data set of rotating 180° horizontally, the threshold value *T* is in the range of 2.8–3.2, and the overall recognition effect of OSKKNN is better.

**Figure 13:**
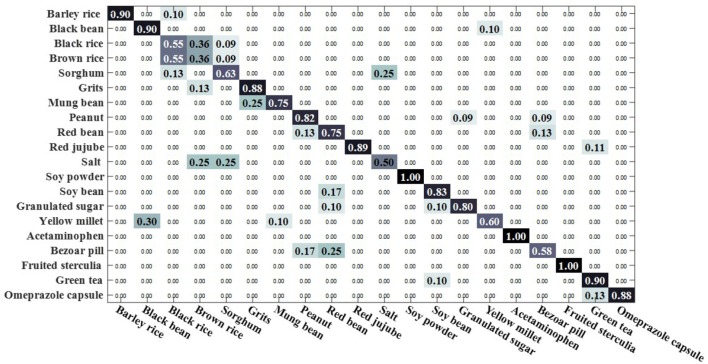
OSKKNN recognition confusion matrix on rotate 180° data when threshold value *T* = 3.3.

**Figure 14:**
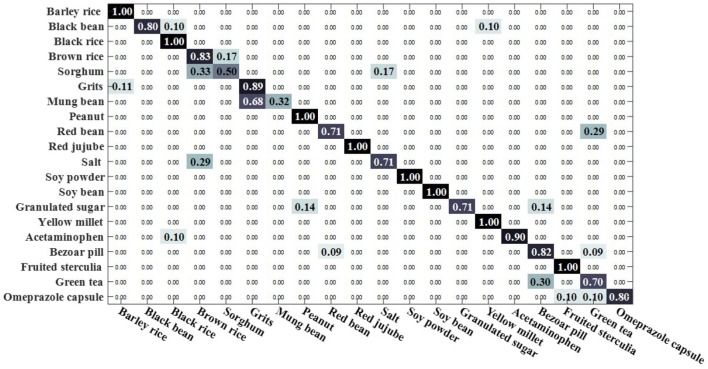
OSKKNN recognition confusion matrix on rotate 180° horizontally data when threshold value *T* = 2.9.

When the threshold value is set to 3.3, the classification confusion matrix of the rotate 180° dataset is obtained as shown in Figure 13, where black rice, brown rice and sorghum are hard to distinguish. 25% of salt was recognized as sorghum, 25% of salt was recognized as brown rice, 30% of yellow millet was recognized as black bean, and 25% of bazaar pill was recognized as red bean. When the threshold value is set to 2.9, the classification confusion matrix of rotate 180° horizontally can be obtained, as shown in Figure 14. Brown rice and sorghum are hard to distinguish. 68% of mung bean was recognized as grits, and the error rate was higher. 29% of red bean was recognized as green tea, 29% of salt was recognized as brown rice, 14% of white sugar was recognized as peanut, 14% of white sugar was recognized as bezoar pill, and 30% of green tea was recognized as bezoar pill. The overall recognition effect is better, and the recognition effect is equivalent in a closed environment. It shows that the method proposed in this paper can solve the problem of recognizing unknown class objects in an open environment and finally recognize all class objects.

#### 5.2.3. Performance Evaluation for Recognizing All Class Objects in Open Environment

The proposed framework mainly addresses the recognition of all class objects in an open environment, so the effectiveness of the method can be assessed using the openness to the overall recognition accuracy. Openness refers to the ratio of known classes and unknown classes, so the factors affecting the accuracy of recognition are the number of unknown classes and the number of known classes. Set 10 classes of known classes and 10 classes of unknown classes, rotate 180° data set at a threshold of 3.3 for experimentation, and rotate 180° horizontally data set at a threshold of 2.9 for experiments.

**Figure 15:**
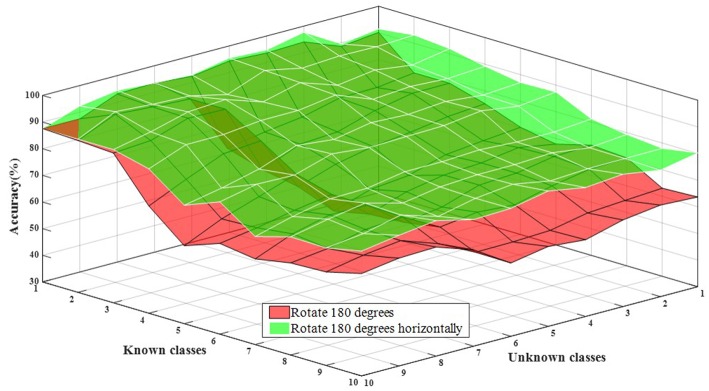
The influence of known classes and unknown classes on the overall accuracy of recognition.

Experimental results are shown in Figure 15. From the test results, it can be concluded that the more known the classes, the worse the recognition effect is. In other words, the recognition accuracy decreases with the increase of the number of known classes, and the performance is stable. For the number of unknown classes, the recognition accuracy of rotating 180° horizontally fluctuates less, while that of rotating 180° fluctuates more. This is because there are some confusing categories and different interaction methods, but a relatively good recognition effect is reached. Therefore, the proposed method can solve the problem of audio recognition for all classes in an open environment.

## 6. Conclusion

With the rapid development of intelligent service robots and the increasing demand, intelligent robots can detect the objects in the container. Because of the limitations of vision and touch, the objects in the container cannot be detected. Therefore, different interaction methods are used to collect the auditory information (sound) for recognizing objects in containers. The experiment proves that the robot can recognize objects in containers well with audio, and the recognition effect is better with the single joint rotation of 180° or horizontal rotation of 180°. It provides a good way for the intelligent service robot to recognize objects in containers when interacting with these containers, which has high application value.

In real life, people are constantly learning and exploring unknowns. Traditional machine learning is carried out in a closed environment, which does not conform to the mode of intelligent robots and people. Therefore, the Kernel-KNN algorithm is improved and extended to solve the problem of audio recognition in an open environment in this paper. Experiments show that the proposed OSKKNN algorithm has a good recognition effect and can solve the problem of using audio to recognize objects in an open environment. It also provides a feasible idea for other fields such as tactile and visual fields.

However, there is still the problem of object confusion in the recognition process, and several classes of object recognition effects are not ideal. Therefore, more economical, simple and convenient multi-modal sensors need to be developed in the future to collect information of multiple modes for better recognition. Develop the more stable, fast and low running cost algorithm. Develop more modal fusion and modal pairing algorithms (Zheng et al., 2019a,b). Combine visual and tactile matching for recognition (Liu et al., 2017, 2018a,b). In the case of reducing the noise of the robot itself, data collection through the integration of multiple interactive actions can improve the recognition accuracy. Recognizing in an open environment is a new research problem that requires more in-depth theoretical research and the development of open recognition methods suitable for all modalities.

## Data Availability Statement

The raw data supporting the conclusions of this manuscript will be made available by the authors, without undue reservation, to any qualified researcher.

## Author Contributions

SJ: contribution was to propose theoretical methods, establish data sets, experiments, and paper writing. HL: contribution was to instruct the theory, experiments and paper writing, and project support. BW: contribution was project support. FS: contribution was to guide paper writing.

### Conflict of Interest

The authors declare that the research was conducted in the absence of any commercial or financial relationships that could be construed as a potential conflict of interest.
